# Gender-Specific Determinants of Instrumental Activities and Associations With Cognitive Decline in TILDA

**DOI:** 10.1093/geroni/igaa057.2295

**Published:** 2020-12-16

**Authors:** Christine McGarrigle, Orna A Donoghue, Joanna Orr, Rose Anne Kenny

**Affiliations:** 1 Trinity College Dublin, Dublin, Ireland; 2 The Irish Longitudinal Study on Ageing (TILDA), Dublin, Dublin, Ireland

## Abstract

Detecting functional decline is critical in recognizing clinical progression into mild cognitive impairment and dementia as people age. This study aimed to investigate differences in Instrumental Activities of Daily Living (IADL) and associations with cognition by gender. Data are from five waves of TILDA, a nationally representative cohort of 8,175 aged ≥50 years, measured every 2 years between 2009 and 2018. IADL assessed were preparing a hot meal, doing household chores, shopping for groceries, making telephone calls, taking medications, and managing money. Cognition was assessed using the Mini-Mental-State Examination (MMSE). Latent class analysis and growth mixture modelling techniques were used to identify IADL and cognitive latent classes and examine change trajectories. IADL trajectories differed by gender and were associated with cognitive decline. Functional decline discrepancies between men and women may be driven by domestic tasks within IADL which are associated with social gender roles, rather than determining differential cognitive trajectories.

